# Supplementary material for: Prostaglandin E2 Induces Skin Aging via E-Prostanoid 1 in Normal Human Dermal Fibroblasts
Source: Int J Mol Sci. 2019 Nov 7;20(22):5555. doi: 10.3390/ijms20225555 (PMC6887779; doi:10.3390/ijms20225555)
Supplement: Supplementary file 1 [file ijms-20-05555-s001.pdf]

Supplementary figure legends

Supplementary Fig. 1

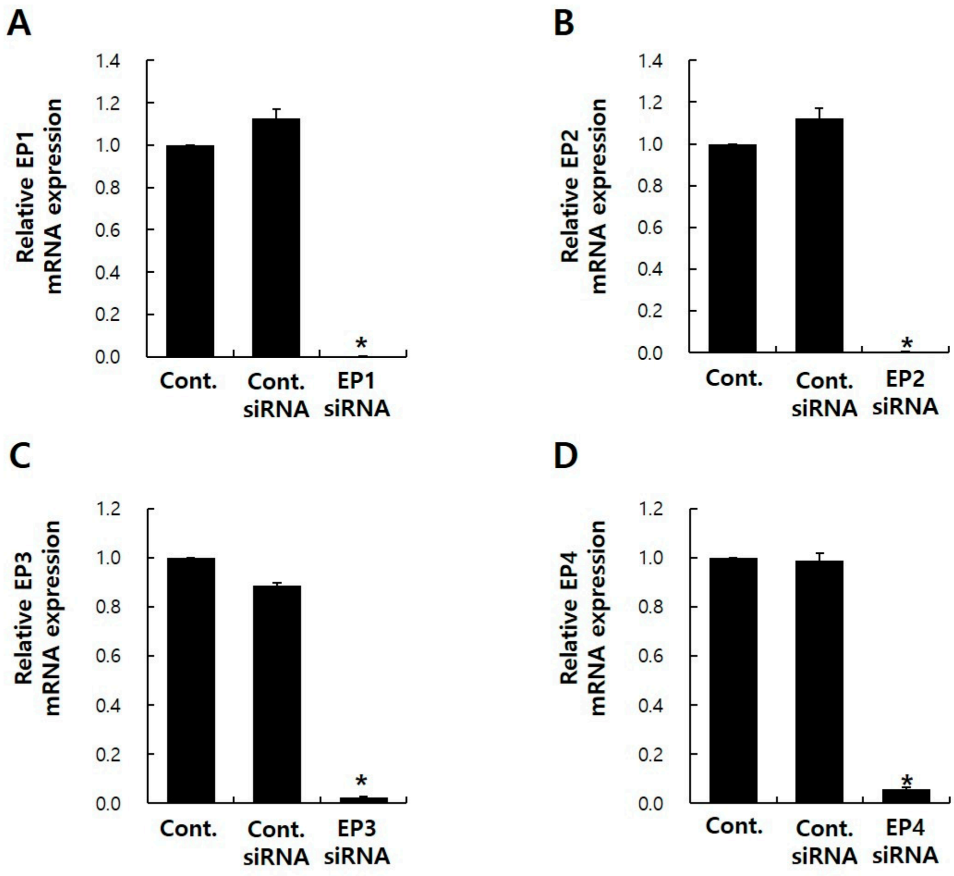

Supplementary Fig. 1 EP1–EP4 siRNAs significantly decrease their own gene expressions. EP1–EP4 siRNA and control siRNA were transfected into NHDFs. Total RNA was extracted, and qRT-PCR was performed for EP1 (A), EP2 (B), EP3 (C), and EP4 (D). Error bars represents the SD of independent experiments. *$P < 0.05$ compared to the control. EP, E-prostanoid; siRNA, small interfering RNA; NHDF, normal human dermal fibroblast; qRT-PCR, quantitative reverse transcription polymerase chain reaction; SD, standard deviation.

Supplementary Fig. 2 (EP2 siRNA)

A

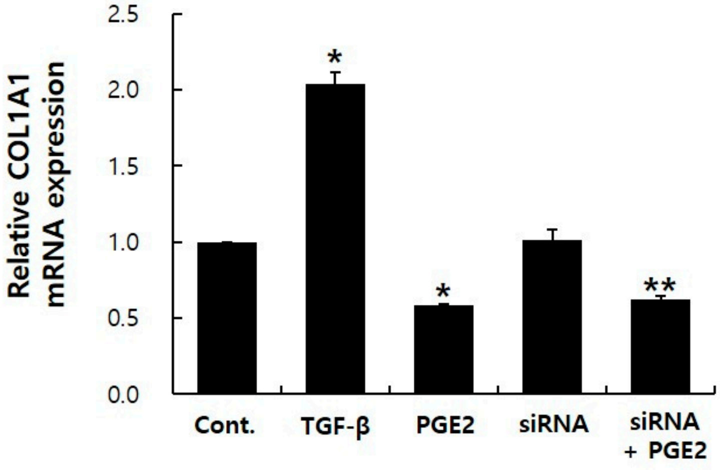

B

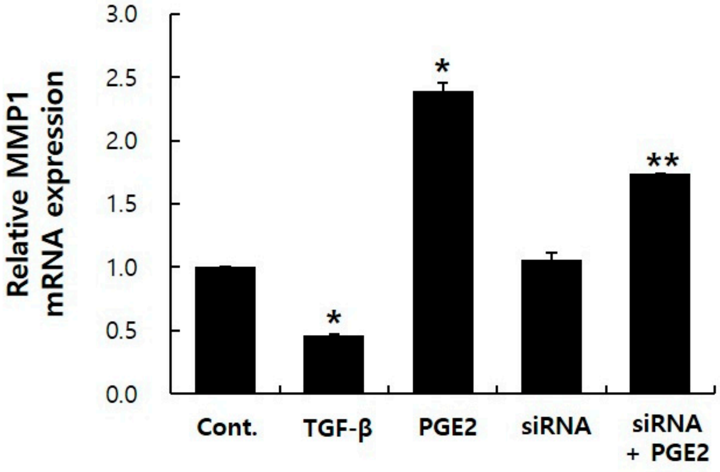

Supplementary Fig. 2 (EP3 siRNA)

C

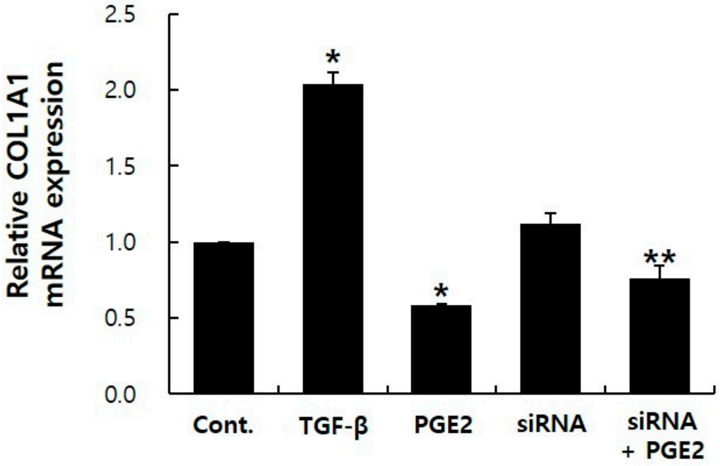

D

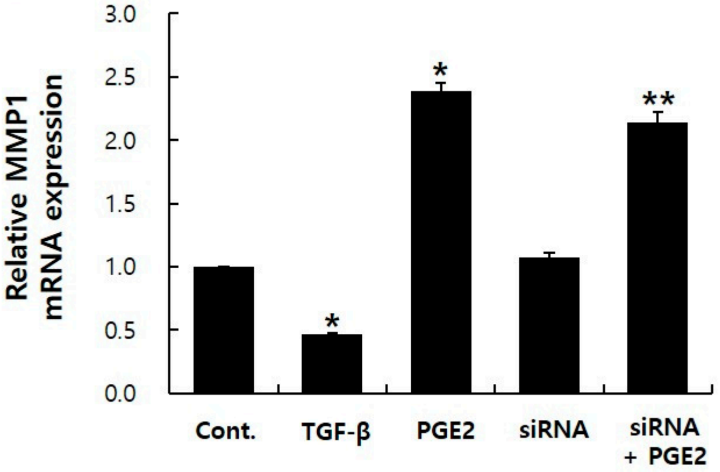

Supplementary Fig. 2 (EP4 siRNA)

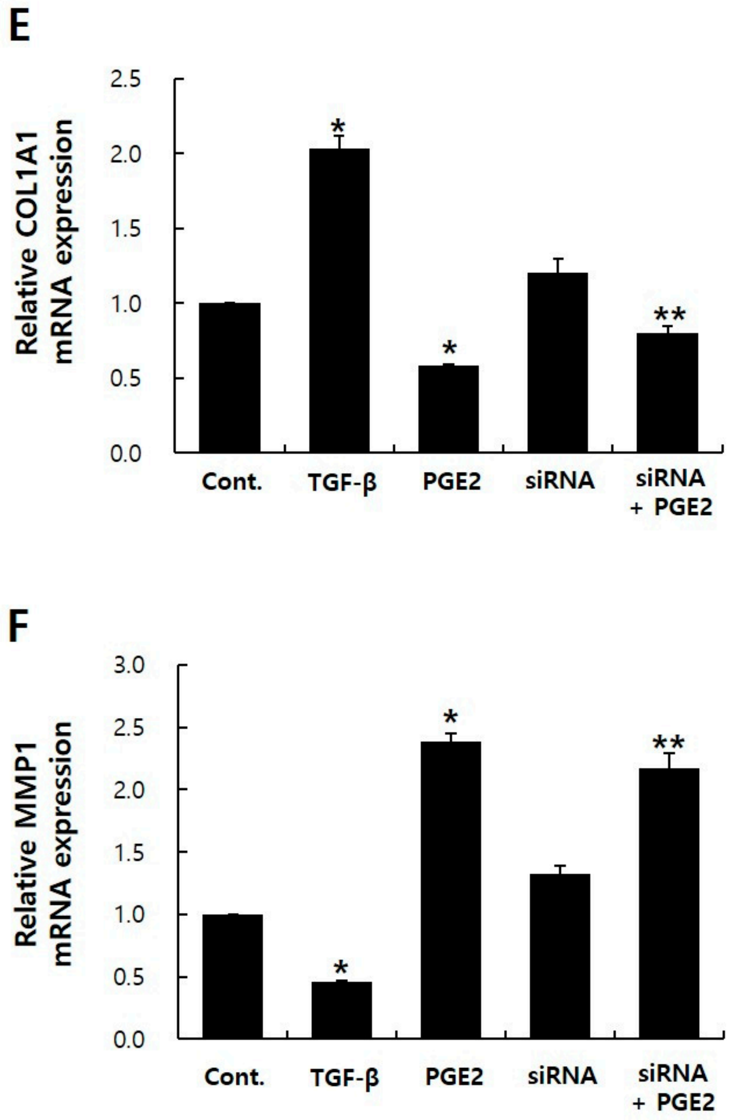

Supplementary Fig. 2 EP2–EP4 siRNAs do not affect COL1A1 and MMP1 expression in NHDFs. EP2–EP4 siRNA and control siRNA were transfected into NHDFs. Total RNA was extracted, and qRT-PCR was performed for COL1A1 and MMP1 (A–F). Error bars represent the SD of independent experiments. *Compared to the control; **$P < 0.05$ compared to siRNA-transfected NHDFs. EP, E-prostanoid; siRNA, small interfering RNA; COL1A1, collagen, type I, alpha 1; MMP1, matrix metallopeptidase 1; NHDF, normal human dermal fibroblast; qRT-PCR, quantitative reverse transcription polymerase chain reaction; SD, standard deviation.
